# Road Sanitation

**Published:** 1904-05-28

**Authors:** 


### The Hospital.

A Journal of

The Medical Sciences and Hospital Administration.

Vol. XXXVI.—No. 922. MAY 28, 1904.

### Road Sanitation.

The quarterly Journal published by the Sanitary Institute frequently contains material of much value and importance to all who are either interested in, or directly responsible for, the conduct of proceedings by which the public health is liable to be affected, either prejudicially or beneficially; and the current number forms no exception to this general rule. Among much other matter of general interest, it furnishes two papers on road and street sanitation which were read at the February meeting of the Institute, and a full report of the discussion upon them. The papers were by Mr. Barber, the Borough Engineer of Islington, and Dr. Parkes, the Medical Officer of Health for Chelsea; and they placed in strong light the magnitude of the evils occasioned by the ordinary dust and mud of our streets and roads, as well as the costliness and difficulty of any effectual measures for dealing with them. Many foot passengers in London streets must from time to time have wondered at the extraordinary abundance of the mud supply, at the mere quantity of it, which, after even a moderate rainfall, is lying in the carriage ways ready to be splashed over clothes, to be carried about on boots, and to be thrown, from time to time, over the faces and into the eyes and mouths of passers-by. Such wonder is diminished when we learn that a cubic foot of what is called "dry" dust, and which is estimated to contain 30 per cent. of water, is converted by a sufficient shower into no less than seven cubic feet of mud, which must be left—as it too often is—to be a public nuisance or must be washed down the gullies, or must be carted away at much expenditure of the labour of men and horses. But, however destructive and annoying may be the mud, the general consensus of opinion is that its raw material, the dust, is much more to be dreaded and condemned. The dust of great cities is largely composed of the dried and powdered material furnished by the excreta of dogs and horses, by domestic offal, and by the refuse thrown out by costermongers and from small shops; and it swarms with bacteria which must often be pathogenetic, which are always likely to be exciting causes of putrefaction, and which find their way everywhere. No doors or windows close in such a manner as to exclude them; and no larder can be in any effective way protected from them. While this applies to towns, and while town surveyors are looking forward to a time when the increasing development of motor vehicles will almost or altogether remove horse traffic and horse droppings from the streets, this same development is rapidly increasing the amount of dust which has to be encountered upon country roads and which is available for distribution in the pantries and dairies of roadside country residences. Such dust, it is true, is granitic in larger, and organic in smaller, proportion than the dust of towns; but even pure granitic dust is not acceptable to the respiratory organs, and there are roads in England which expose automobilists to dangers analogous to those which attend upon manufactures in which "dry grinding" is required. Dust-producing industries and vehicles may be absolute necessities of our civilisation, but, nevertheless, they resemble "caller herrin'," in being also "lives o' men."

In this case, as in so many others, it is more easy to point out an evil than to suggest a practical remedy. As far as towns are concerned the united wisdom of surveyors is only able to say that cleansing costs much money, that the road surfaces least liable to be broken into dust are often slippery and dangerous for horses, that the speedy disappearance of the town horse and of the town dog is greatly to be desired, that even the "necessary" cat is not so "harmless" as he or she has been called, that a perfect sanitary system would imply the effective washing away of dust once, if not twice, in the 24 hours, and that hence, if the public want clean and wholesome streets, the public must be prepared to pay for them, and must not regard increased rating with the amount of disfavour which it now commonly excites. An abundant supply of water for street use is declared to be essential to improvement, and additions of various chemicals to this water, additions rather suggestive of Sir John Simon's scornful reference to "vague chemical libations and powderings," are not without their advocates. Mention has been made, moreover, of the possible application to road and street surfaces of some tarry or oily material by which the formation of dust would be greatly hindered, and, although nothing very definite has yet been said under this division of the subject, there is enough to recall the trials of "Westrumite," concerning which a few brief and insufficient, but generally favourable, notices have appeared in the columns of one or two newspapers. Some of our readers may remember an experiment which was conducted, now a good many years ago, in the borough of Paddington, and which was said at the time to be exceedingly successful. The streets were watered, during nearly the whole of one summer, with water holding in solution what were called, from the name of the patentee, "Cooper's Salts," and which were, we believe, a mixture of equal parts of chloride of calcium and chloride of sodium. The general result was excellent, for in dry weather the saline material formed a sort of cake over the road surfaces which did not readily crumble into powder, and which so quickly absorbed moisture from the atmosphere that it was seldom or never quite dry. The shopkeepers in the Harrow Road declared that the diminution of dust greatly diminished the injury previously suffered by goods exposed for sale; and the chlorides appeared to exert a distinctly antiseptic influence upon organic matter. Notwithstanding these advantages, the use of the salts was discontinued, in consequence, we believe, of some uncertainty concerning the rights of the patentee; but it seems probable that by this time any such question must be at an end, and that other trials in the same direction might be instituted. Something of the same effect would possibly be produced by that watering with sea-water which has more than once been proposed for inland towns, but never, so far as we know, carried into effect. It is evident that reforms in the present methods of street cleansing are called for, and the whole question deserves the attention of enlightened municipalities.

